# AI-assisted [^18^F]FDG PET/CT quantification: a time-saving approach in large B-cell lymphoma?

**DOI:** 10.1186/s40658-026-00923-y

**Published:** 2026-08-16

**Authors:** Virgile Micheletti, Étienne Marchal, Maxence Gambiez, Laurent Dercle, Marc-Etienne Meyer, Xavier Palard-Novello, Antoine Girard

**Affiliations:** 1 Department of Nuclear Medicine, Amiens-Picardy University Hospital, 1 Rue du Professeur Christian Cabrol, Amiens, France; 2 Department of Radiology, New York Presbyterian Hospital - Columbia University Irving Medical Center, New York, NY 10039 USA; 3 Department of Nuclear Medicine, Eugène Marquis Center, Rennes, France; 4 INSERM, LTSI-UMR 1099, University of Rennes, Rennes, France

**Keywords:** Lymphoma, Artificial intelligence, Deep learning, TMTV, Dmax, Inter-reader agreement

## Abstract

**Purpose:**

To assess if AI-assisted segmentation on [^18^F]FDG PET/CT significantly reduces time in quantifying Total Metabolic Tumor Volume (TMTV) and the greatest distance between two lesions (Dmax) in Large B-cell lymphoma (LBCL) patients.

**Methods:**

A retrospective study at two institutions included 56 LBCL patients (Ann-Arbor staging: 14% with no disease, 25% stage I-II, 61% stage III-IV). Two methods were compared: AI-assisted segmentation (Pionus^®^ v1.1.0, PAIRE, Paris) automatically identified lesions with expert correction of AI errors, while expert-led segmentation was fully manual. Both methods used a 41% SUVmax threshold.

**Results:**

AI-assisted segmentation was 41% faster, saving an average of 104 s per patient (152 ± 134 s vs. 255 ± 270 s, *p* < 0.001), with time saved correlating with TMTV (r = 0.67, *p* < 0.001). No significant differences in TMTV or Dmax were found between methods. Intraclass correlation coefficients were high for both TMTV (0.91 and 0.95) and Dmax (0.97 and 0.95), with strong inter-reader agreement.

**Conclusion:**

AI-assisted segmentation significantly reduces time for TMTV and Dmax measurement in LBCL patients, especially in patients with high tumor burden, while maintaining high reliability and inter-reader agreement. The impact on the integrated clinical workflow has yet to be assessed.

## Introduction

The contribution of algorithms based on artificial intelligence (AI) and deep learning (DL) is growing rapidly in medicine. In the field of nuclear medicine imaging, it has already proved its worth, notably in the optimization of injected radiopharmaceutical activity and/or acquisition time thanks to a denoising algorithm based on deep learning [1, 2]. Other DL-based solutions have also been commercialized and are tending to be implemented in clinical practice, such as automatic lesion detection and segmentation algorithms. These tools are needed to save medical time at a time when, worldwide, the number of PET scans performed each year is growing much faster than the population of nuclear physicians. The integration of these algorithms into the clinical workflow is hampered by the low level of evidence concerning their accuracy, reliability and reproducibility in the wide variety of clinical situations encountered in daily practice [3, 4].

Due to the prevalence of the disease and the often high tumor burden, DL-based detection and segmentation algorithms are presumed to be particularly useful for the analysis of [^18^F]FDG PET/CT imaging of lymphoma patients [5–7]. In large B-cell lymphoma (LBCL), the use of [^18^F]FDG PET/CT is well established for staging and assessing response to therapy [8]. Two quantitative metabolic features have been reported as prognostic factors: total metabolic tumor volume (TMTV) and distance between the two most distant lesions (Dmax) [9–13]. Manual measurement of these features is time-consuming and currently lacks inter-observer reproducibility [14, 15]. Assessing reliability of DL-based algorithms for measuring TMTV and Dmax is a prerequisite for implementing their routine use in clinical workflow. The amount of medical time that can be saved also remains to be assessed.

The aim of this study was to assess the reproducibility of PET features for tumor burden (TMTV) and spread (Dmax) when obtained using a commercially available DL-based solution for lesion detection and segmentation. We also evaluated the potential savings in medical time spent taking these measurements.

## Material and methods

### Study population

We retrospectively analyzed patients who underwent [^18^F]FDG PET/CT as part of their pre-CAR T Cells workup for LBCL in two different French institutions between October 2018 and October 2021 [16]. Non-inclusion criteria were people under guardianship or curatorship or deprived of liberty.

### FDG PET acquisition protocol

Six hours of fasting and a blood glucose level below 12 mmol/L were required before intravenous injection of [^18^F]FDG. PET acquisitions were performed 1 h after injection.

### FDG PET image analysis

Each PET was pseudonymized and then analyzed separately by two nuclear medicine physicians (R1: VM and R2: EM) using LIFEx v7.2 software [17]. The lesions suspected to be related to LBCL were identified and segmented by two different methods at least three months apart by each physician. Two parameters were collected for both readers, with both methods: the TMTV (in mL) defined as the sum of every individual lesion metabolic volume (MTV), and the Dmax (in cm), defined as the distance separating the two most distant lesions. Additionally, for both methods, the time required for the whole process was recorded for reader #1 from the opening in the viewer of PET/CT images and volumes delineated by the solution.

### Expert-led segmentation

The first method consisted in manually selecting lesions and applying a segmentation threshold of 41% of the SUVmax of each lesion, in accordance with the recommendations of the EANM [18]. This method was considered as the current state of the art.

### AI-assisted segmentation

With the second method, readers first used a DL-based algorithm (Pionus v1.1.0 by PAIRE, Paris, France) to detect and delineate lesions, and subsequently manually deleted volumes that were not considered to be lesions related to LBCL and/or manually added lesions considered missed by the solution. The same threshold of 41% of the SUVmax of each lesion was applied for tumor delineation.

The studied CE-marked solution (Pionus ^®^ v1.1.0, PAIRE, Paris, France) uses a 3D deep convolutional neural net-based AI to detect lesions in [^18^F]FDG PET/CT scans based on the nnU-net architecture. It underwent a comprehensive training regimen, encompassing a total of 4993 studies for its training phase [7]. For its training, all lesions including both inflammatory (infectious or granulomatosis) and tumoral uptakes were included without distinction.

### Statistical analysis

The primary evaluation endpoint was the time it took the physician to detect and delineate the entire tumor volume, eventually correcting failed or incomplete segmentations, and excluding non-tumor uptake (image reconstruction and transfer operations, AI processing, and software export and import were not included in the recorded time). Statistical tests were performed using MedCalc software version 12.5.0.0 (© MedCalc Software Ltd). The distributions of quantitative values were presented with the mean and the standard deviation (mean ± SD). Because of the non-normal distribution of the features, the TMTV, the Dmax and the measurement were pairwise compared using the Wilcoxon signed-rank test. Comparisons of proportion were performed with the Chi-squared test. The correlations of TMTV and Dmax between both methods were evaluated in the sample with the Spearman correlation coefficient (r). The inter-reader agreements were calculated with the intraclass correlation coefficients (ICC) of absolute agreement for average measures. The statistical tests were two-sided, and their results were considered statistically significant if *p* < 0.05.

## Results

### Sample characteristics

Fifty-six [^18^F]FDG PET/CT of 56 patients from two French institutions were analyzed retrospectively (46 from the Eugène Marquis Center of Rennes and 10 from the University Hospital of Amiens Picardy). Patients and PET/CT characteristics are detailed in Table 1.

**Table 1:** Patients and PET/CT characteristics

Number (%) or average (± SD)	
Patients (n = 56)	
Sex (male)	36 (64%)
Age	60.2 (± 11.5)
Body mass index	25,2 (± 5.6) )
Stade Ann-Arbor	
No disease	8 (14%)
I—II	14 (25%)
III—IV	34 (61%)
PET/CT procedure	
Injected activity	223 (± 58)
Injection-acquisition delay (min)	71 (± 11)
Blood glucose level (mmol/L)	5.65 (± 0.9)
PET/CT system	
Siemens Biograph mCT	41 (73%) 12 (21%) 12
GE Discovery MI	12 (21%) 4
Siemens Biograph Vision	2 (4%) 1(
Philips Vereos	1 (2%)

### Evaluation of time-saving

**Fig. 1:**
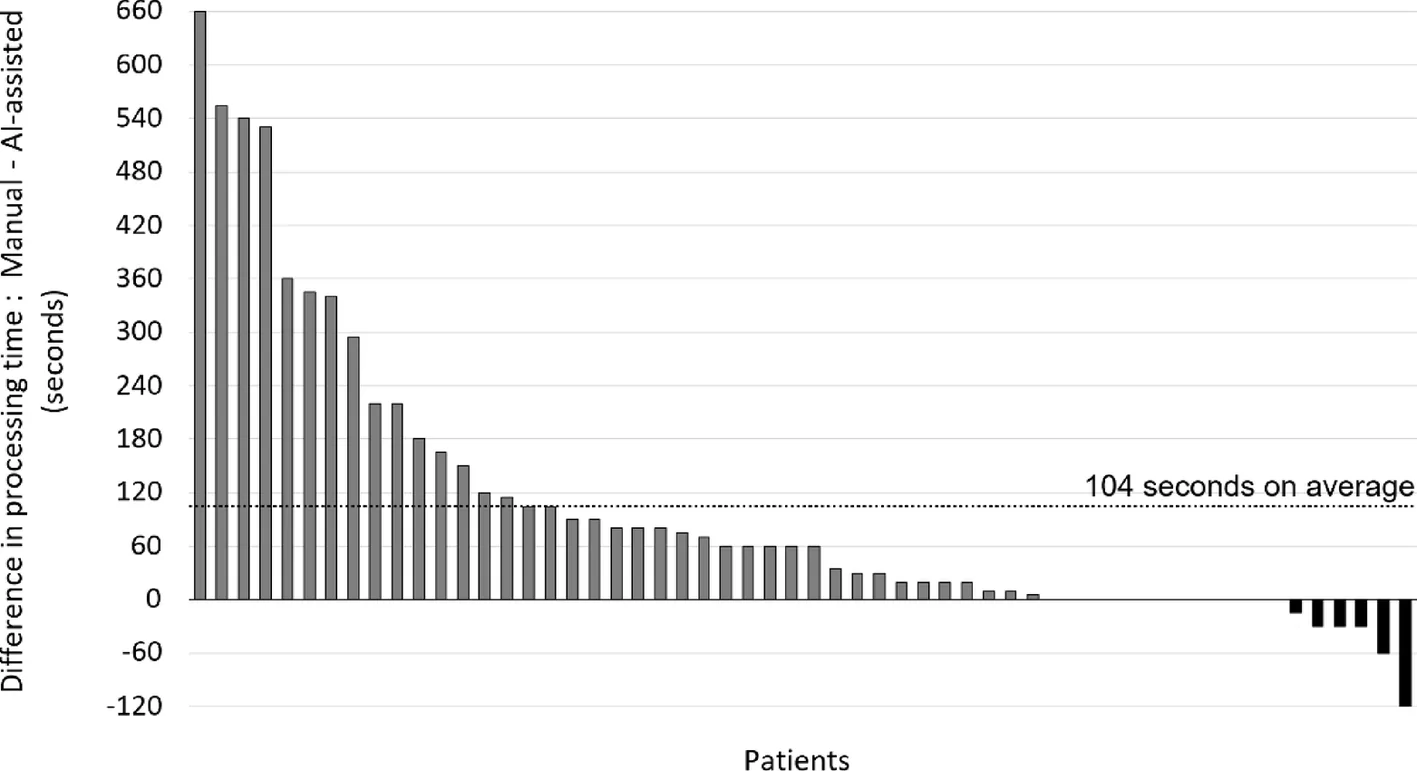
Difference of time spent between manual delineation and delineation performed with the help of AI, in seconds. The difference is shown in grey when the AI-assisted method is faster than the full-manual method, and in black otherwise. The dotted line represents the average time that would have been spared using the DL-based solution

**Fig. 2:**
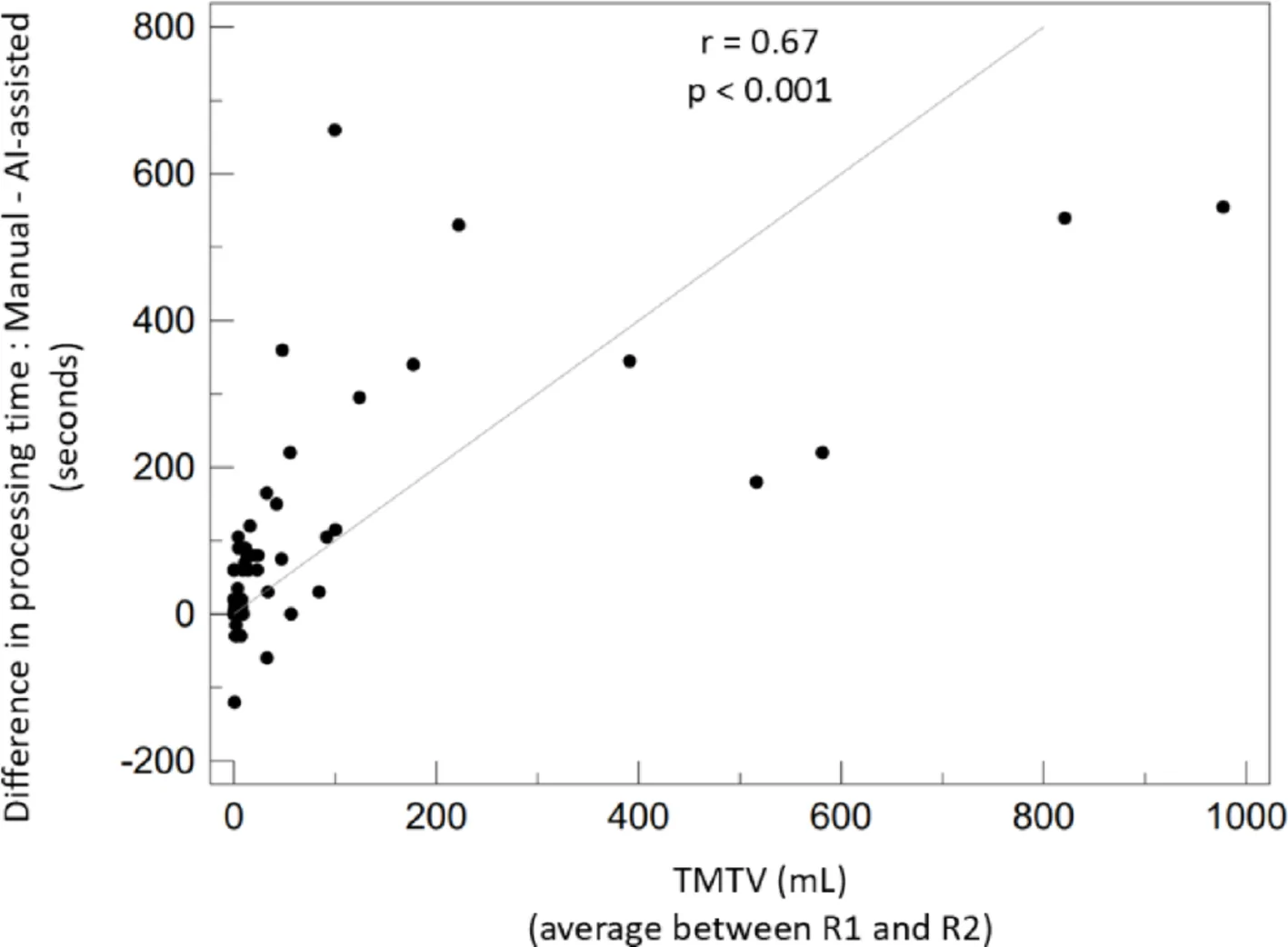
Correlation between the time that would have been spared using the AI-assisted segmentation and the tumor burden of patients

The mean time for AI-assisted delineation of lesions (152 s ± 134) was significantly shorter than for the same task performed fully manually (255 s ± 270, *p* < 0.001) (Fig. 1). On the scale of this sample of 56 patients, 1.61 h of medical time would have been saved with the use of the solution, corresponding to a mean of 104 s per patient. The time-saving was moderate to strongly correlated to the TMTV (r = 0.67, *p* < 0.001) (Fig. 2).

### Comparison of TMTV measurements

**Fig. 3:**
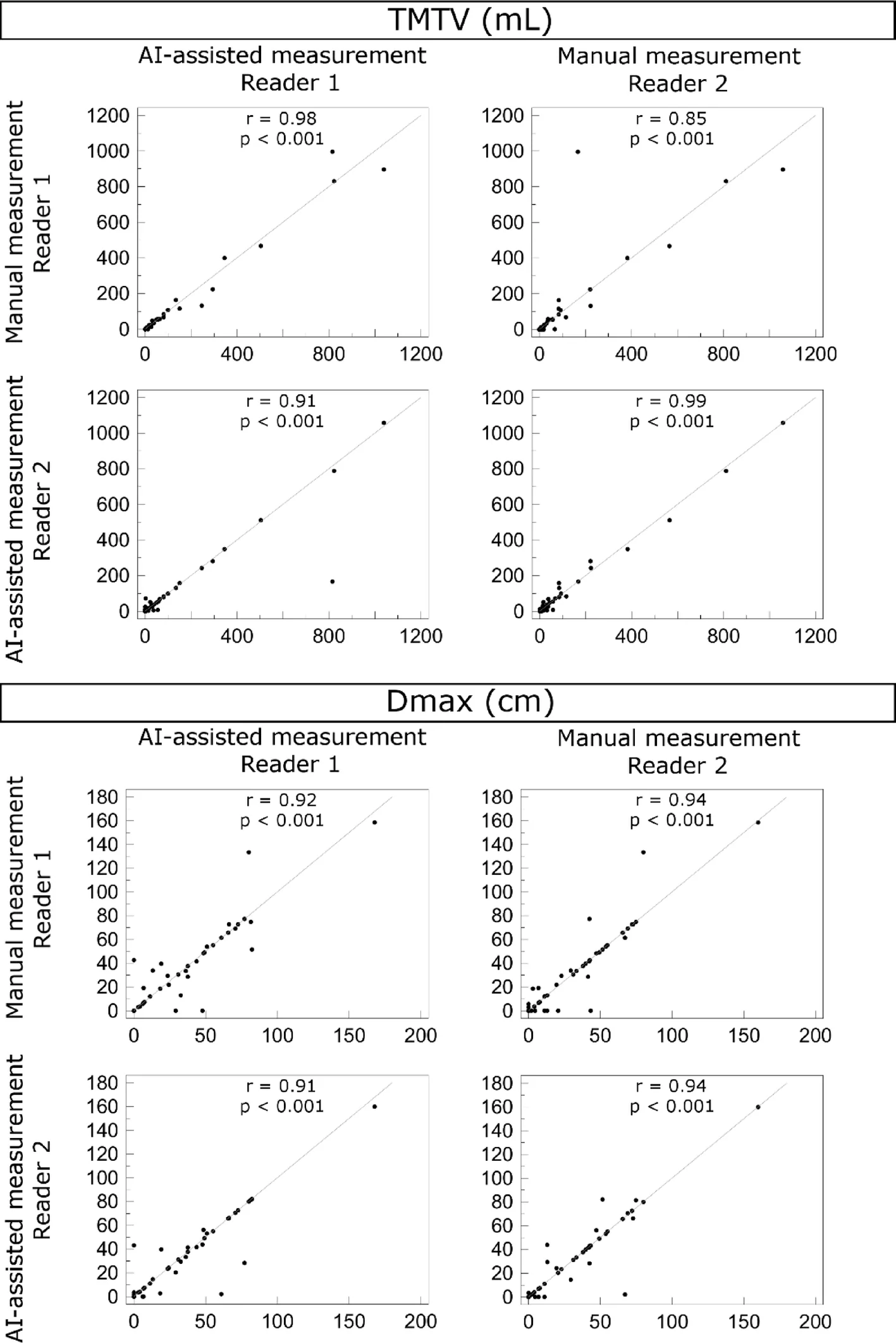
Correlation between TMTV and Dmax measurements across different readers and different methods (fully manual method and method with readers assisted by an AI-based solution)

**Fig. 4:**
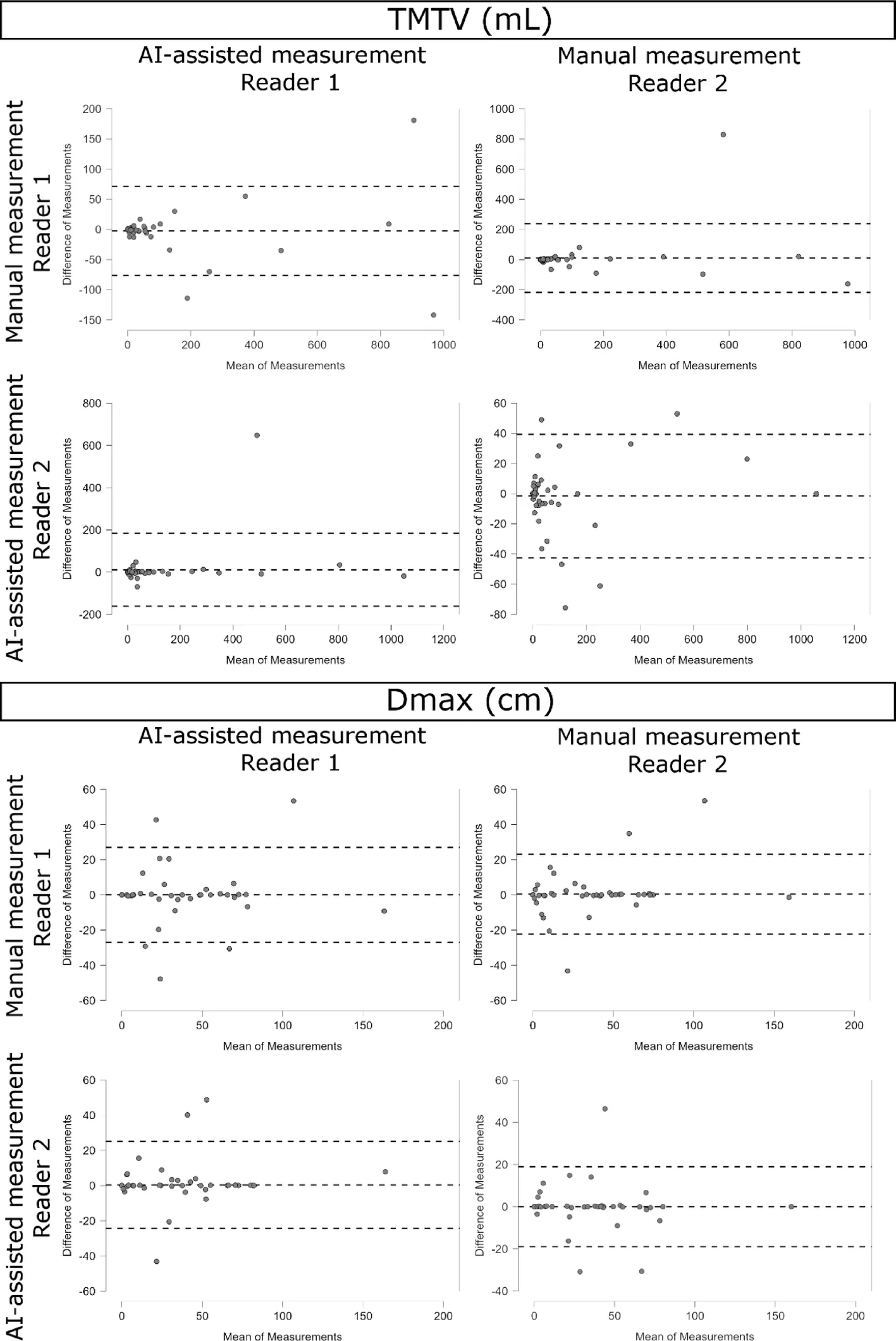
Bland–Altman plots between TMTV and Dmax measurements across different readers and different methods (fully manual method and method with readers assisted by an AI-based solution)

**Fig. 5:**
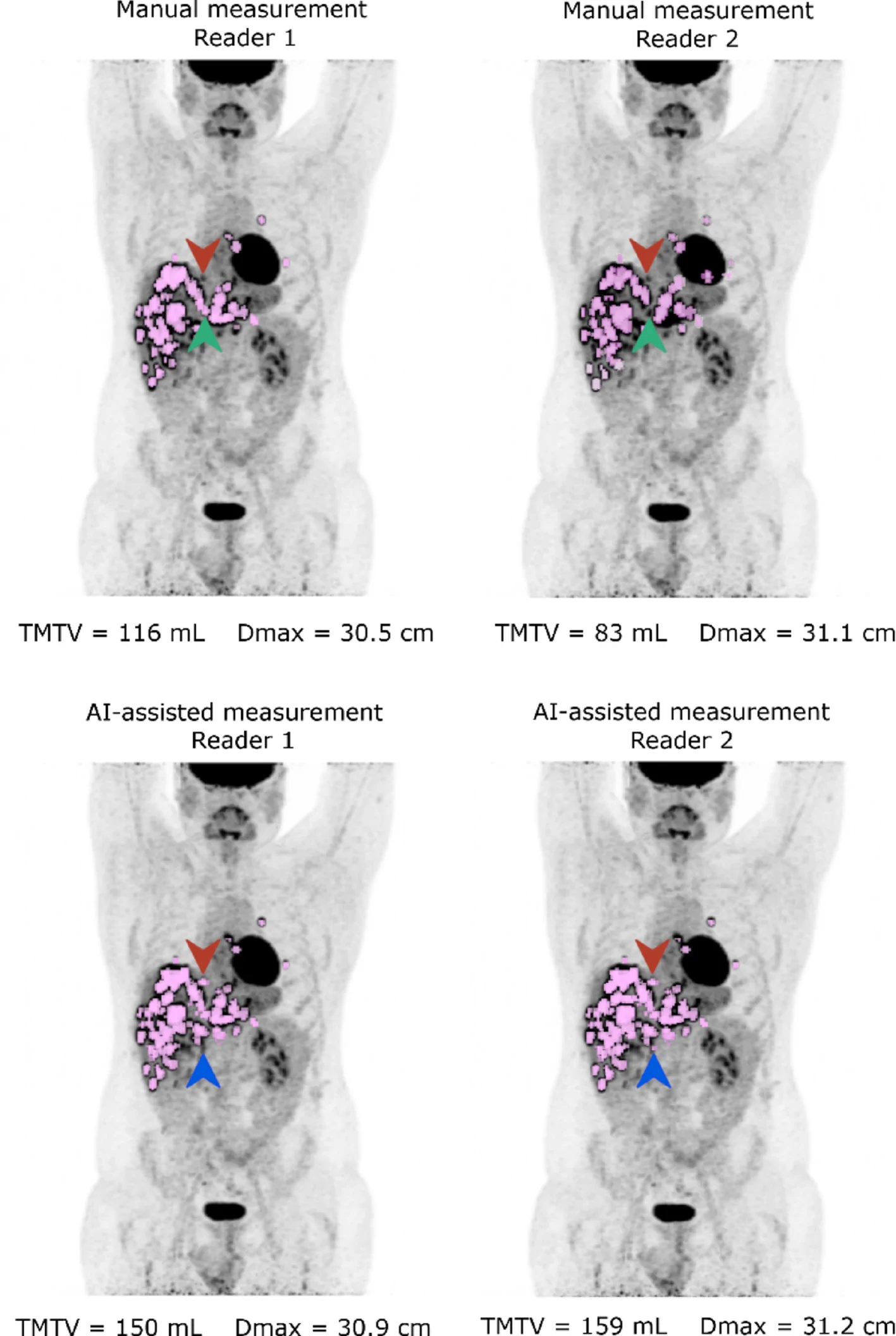
Example of TMTV and Dmax measurement by both readers with both methods (manual and assisted by the DL-based solution). Red arrowheads indicate a lesion detected with the help of the AI-based solution, but not by humans alone. Green arrowheads indicate a lesion detected manually only by reader 1, and blue arrowheads a lesion detected with the help of AI only by reader 2

There was no statistically significant difference between TMTV measured manually (R1: median = 9.0 mL [range = 0.0 mL, 996.0 mL]; R2: median = 11.5 mL [range = 0.0 mL, 1058.0 mL]) and with the AI assistance (R1: median = 10.0 mL [range = 0.0 mL, 1039.0 mL]; R2: median = 7.9 mL [range = 0.0 mL, 1058.0 mL]) when pairwise compared for each reader (R1: *p* = 0.49; R2: *p* = 0.62). TMTV measured manually and with the AI assistance were highly correlated for both readers (R1: r = 0.98 [95%CI = 0.97, 0.99]; R2: r = 0.99 [95%CI = 0.99, 1.00]). (Figs. 3 and 4). An example of TMTV measurement by both readers using both methods is shown in Fig. 5.

### Comparison of Dmax measurements

There was no statistically significant difference between Dmax measured manually (R1: median = 9.7 cm [range = 0.0 cm, 158.6 cm]; R2: median = 12.1 cm [range = 0.0 cm, 160.0 cm]) and with the AI assistance (R1: median = 9.3 cm [range = 0.0 cm, 167.8 cm]; R2: median = 9.2 cm [range = 0.0 cm, 160.0 cm]) when pairwise compared for each reader (R1: *p* = 0.78; R2: *p* = 0.79). Dmax measured manually and with the AI assistance were highly correlated for both readers (R1: r = 0.92 [95%CI = 0.86, 0.95]; R2: r = 0.94 [95%CI = 0.89, 0.96]). (Figs. 3 and 4). An example of Dmax measurement by both readers using both methods is shown in Fig. 5.

### Comparison of inter-reader agreement

There was no statistically significant difference of ICCs for inter-reader agreement between manual and AI-assisted measures of TMTV (0.91 [95%CI = 0.85, 0.95] and 0.95 [95%CI = 0.92, 0.97], respectively) and Dmax (0.97 [95%CI = 0.95, 0.98] and 0.95 [95%CI = 0.92, 0.97], respectively), that were all very high.

## Discussion

The prognostic value of TMTV and Dmax on [^18^F]FDG-PET images in LBCL patients has been demonstrated by several studies [9–13, 16, 19–23]. However, their widespread use in daily practice is hampered by the time required to delineate lesions, particularly in patients with large tumor burdens. In this study, we examined whether the use of a commercialized DL-based solution can help save medical time on this task, and its impact on values and inter-reader reproducibility for TMTV and Dmax measurements. In our sample, the use of such a solution required on average 1 min and 44 s less time for the same task, with high reproducibility between the two readers when compared to the full manual method.

In this study, TMTV and Dmax were not statistically different when measured manually (current reference method) or using the DL-based solution. Intra-reader agreement—with and without the solution—was very high for TMTV (r = 0.98 and 0.99, respectively for readers 1 and 2) and Dmax (r = 0.92 and 0.94, respectively for readers 1 and 2). Moreover, the challenged method (physician assisted by the AI-based solution) did not introduce any more variability in the measurements (TMTV and Dmax), compared to the refence method, than the pre-existing variability among the physicians performing these measurements. Therefore, we can consider that the implementation in daily practice of such a solution to assist the physician in measuring TMTV and Dmax in LBCL patients would not hinder the application of previously published prognostic thresholds, based on studies using a fully manual measurement method. We should point out that in the present study, the DL-based solution was not evaluated on its own, but as an aid to the physician. In previous studies evaluating the same type of solution working alone, most reported a significant difference in TMTV compared with the reference method. Testing the DL-based solution in LBCL patients without human correction, Blanc-Durand et al. reported an underestimation of mean TMTV of up to 116 ml (20.8%) [7], and Capobianco et al. found a significant but moderate to strong correlation coefficient for TMTV (ρ = 0.76) [5]. Revailler et al. performed better in training a home-made fully convolutional neural network VNET, reaching a correlation of r = 0.92 with the TMTV measured manually [24]. In contrast, when integrating commercial solutions as a tool to complement human reading, Karimdjee et al. reported a very high intra-class coefficient of 0.999 for TMTV compared with the reference method [6]. In a previous study, our team evaluated the diagnostic accuracy (sensitivity, specificity, positive predictive value, and false-positive regions) of the AI-based solution to detect neoplastic lesions (lymphomas and melanomas), both when used on its own and as a diagnostic aid for physicians [25].

The inter-reader agreement observed was high to very high with the reference method for TMTV (r = 0.85) and Dmax (r = 0.94), and was not significantly affected by the use of the AI base solution (r = 0.91 for TMTV and Dmax). One patient had very different TMTVs reported by readers 1 and 2, irrespective of the method used. This patient was suspected of having peritoneal carcinosis, for which the interpretation differed from one physician to the other, one considering diffuse involvement of the peritoneum while the other considered only multifocal lesions without diffuse involvement. They were both consistent with themselves, regardless of the method. In this context where both readers applied the same clear instructions regarding tumor delineation, the additional use of an automated DL-based solution did not appear to reduce variability between readers. However, in general, the method used to measure TMTV varies considerably and needs to be standardized [26]. DL-based algorithms could be useful tools in this respect. The segmentation threshold applied can also be a source of variability. In the present study, we choose to apply a threshold of 41% of the SUVmax, in accordance with the current EANM guidelines, even if this value does not rely on strong evidences.

In our sample, accurate tumor detection and delineation required on average 104 s (41%) less with the DL-based solution than the same task performed entirely manually. The time saved was particularly high in patients with larger tumors. Interestingly, for 6 PET/CT scans, processing took longer with the solution than manually, with a difference of up to 120 s. The patients concerned had little or no tumor burden (Figs. 1 and 2). The incremental time was mainly spent manually identifying and removing false-negative volumes of interest detected by the solution. It should be noted that reading times were recorded for only one reader, without taking into account any variations in efficiency among readers, which constitutes a significant limitation of this study. In a previous study with a sample including 51 [^18^F]FDG PET/CT scans of patients with diffuse LBCL with larger TMTV (mean = 285.4 cm^3^), Karimdjee, et al. reported a mean time of 150 s saved per measurement with automated method manually corrected compared to manual detection and delineation [6].

With these DL-based solutions reducing the medical time required to accurately measure TMTV and Dmax, the use of these well-documented prognostic factors will be easier, facilitating their widespread use in clinical studies and daily practice. It should be borne in mind that this study involved a homogeneous LBCL sample of just 56 patients from two institutions. Thus, the transposition of our results to other types of population needs to be validated by further studies. Nevertheless, the PET/CT examinations in the present sample were performed under real-life conditions on several types of PET/CT systems, ensuring the robustness of our results in the face of practice variability.

## Conclusion

In this study, we evidenced that the use of a commercially available DL-based solution, such as Pionus ®, detecting and delineating tumors on [^18^F]FDG PET/CT images, before the physician’s expertise could spare a significant amount of medical time for TMTV and Dmax measurement in patients with LBCL. And this, with a very high inter-reader agreement without any difference compared to the manual method of reference. However, the impact of a fully integrated clinical workflow on this time-saving method has yet to be evaluated.

## Data Availability

The datasets generated during and/or analyzed during the current study are available from the corresponding author upon reasonable request.
